# A Decade of Marketing Authorization Applications of Anticancer Drugs in the European Union: An Analysis of Procedural Timelines

**DOI:** 10.1007/s43441-021-00260-5

**Published:** 2021-02-04

**Authors:** Marjolein Garsen, Maaike Steenhof, Alex Zwiers

**Affiliations:** Zwiers Regulatory Consultancy, Pivot Park, Kloosterstraat 9, 5349 AB Oss, The Netherlands

**Keywords:** Cancer, Drug development, EMA, Accelerated assessment, Conditional marketing authorization, Scientific advice

## Abstract

**Background:**

Cancer is a serious global health problem and a major cause of death. The European Medicines Agency (EMA) has established several regulatory initiatives to expedite the development and authorization of drugs to ensure timely access of patients. In this study, we analyzed the procedural timelines of marketing authorization applications for anticancer drugs in the EU, with a specific focus to special regulatory programs, scientific advice and company size.

**Methods:**

Anticancer drugs that received an opinion from the EMA between January 2010 and December 2019 were included in the study. Public assessment reports were used to obtain publicly available information of the drugs.

**Results:**

We identified 96 applications for new anticancer drugs. 34 applications were granted access to at least one expedited program offered by the EMA. Total procedure time was reduced from average 370 to 200–215 days when accelerated assessment was granted. Granting of a conditional marketing authorization or an orphan designation, as well as having scientific advice, only mildly affected total procedure time. Average total procedure time of small companies was much longer compared with medium-sized and large companies (483 versus 356 days), which was caused by an increased clock stop time.

**Conclusion:**

Total procedure time for anticancer is mainly affected by the granting of accelerated assessment, which reduced the total procedure time, and company size, where total procedure time is much longer for small companies. Small companies are advised to have, and especially adhere to scientific advice to reduce procedure time and increase the chance of success.

**Supplementary Information:**

The online version contains supplementary material available at 10.1007/s43441-021-00260-5.

## Introduction

Cancer is a serious global health problem and a major cause of death. In 2018 the World Health Organization (WHO) reported ~ 18 million new cases of cancer and ~ 10 million deaths from the disease worldwide, and the incidence of cancer is still increasing [1]. Over the years many big advances in the prevention, diagnosis, treatment and cure of cancer have been made, which have resulted in a 27% decline in cancer death rates since the peak in 1991, an increased five year survival, and an increased number of registrations of anticancer drugs [2]. Despite all recent advances in the treatment of cancer, there are still many types of cancer that remain hard to treat. The Belgian National Institute for Health and Disability Insurances on a yearly basis publishes an unmet medical need list [3]. In 2020, this list contains 66 diseases of which 37 are cancers. As long as cancer cannot be cured, there will be a need for new therapeutic advances for the treatment of cancer.

Cancer medicines in the European Union (EU) are evaluated by the European Medicines Agency (EMA) under the centralized procedure. Evaluation of a marketing authorization application (MAA) by the Committee for Medicinal Products for Human Use (CHMP) of the EMA can take up to 210 days, excluding clock stop times when applicants have to provide additional information. In the interest of public health, the EMA has established regulatory initiatives to expedite the development and authorization of drugs with promising efficacy and potential to fill unmet medical needs for patients [4]. In 2006 the EMA implemented the conditional marketing authorization (CMA). A CMA may be granted if the CHMP finds that the benefit-risk balance is positive, an unmet medical need will be fulfilled, it is likely that the applicant will be able to provide comprehensive data, and the benefit to public health on the immediate availability of the medicinal product outweigh the risks due to need of additional data [5]. Another initiative to expedite the authorization of drug is the implementation of accelerated assessment (AA) for drugs of major public health interests, in particular therapeutic innovations, which reduces the evaluation time of the CHMP from 210 to 150 days [6]. At any stage of the development of a drug a developer can ask scientific advice (SA) from the EMA on the best methods and study designs to generate robust data on the quality, efficacy and safety of a drug and avoid major objections regarding study design [7]. This is particularly helpful for small and medium enterprises (SMEs) who may have limited knowledge of the regulation of medicines [8]. SA also plays an important role in two other initiatives of the EMA, priority medicines (PRIME) and orphan designation (OD). PRIME has been launched in March 2016 to enhance support for the development of medicines that target an unmet medical need and is based on enhanced interaction and early dialogue between developers and EMA to optimize development plans and speed up evaluation so that these medicines can reach patients earlier [9]. OD is implemented by the EMA to encourage the development of medicines for rare diseases. Companies may benefit from protocol assistance, a type of SA specific for designated orphan medicines [10].

Over the last decade there has been a significant increase in registrations of anticancer drugs in the EU. Despite the regulatory initiatives of the EMA to expedite the development and authorization of anticancer drugs, marketing authorization and market access in the EU is slower than in the USA, where patients get access to new products earlier [11, 12]. The aim of this study was to define the duration of the evaluation of MAAs for anticancer drugs in the EU in the period 2010 to 2019. In particular, we examined the effect of special regulatory programs, SA and company size on total procedure time, and highlighted the differences among them based on biological properties.

## Methods

The human medicine highlights published by EMA (https://www.ema.europa.eu/en/news-events/publications/newsletters) were used to identify oncology products with a positive or negative CHMP opinion between January 2010 and December 2019. Products under the headings cancer, positive CHMP opinions on new medicines and negative CHMP opinions on new medicines in the human medicine highlights were analyzed to evaluate whether they met the following criteria to be included: (i) article 8(3) full or full-mixed application as legal basis; (ii) new active substance; and (iii) products developed for the treatment of the cancer (products developed for treatment of symptoms caused by cancer or cancer treatment, e.g., treatment of pain caused by cancer, treatment of nausea and vomiting caused by chemotherapy, or treatment of chemotherapy-induced neutropenia, were excluded). A quality check (double entry of products) was performed by a second investigator to ensure that all oncology products fitting the criteria as described above were included.

For all oncology products that were included, the publicly available European Public Assessment Report (EPAR) of the initial marketing authorization on the website of the EMA was used to obtain public information of the medicines. In case of a negative opinion, the EPAR-refusal-public-assessment-report was used (further referred to as EPAR). From the EPARs we extracted general information (marketing authorization holder (MAH) name, type of product (small molecule or biotech-derived product), active substance, and therapeutic indication), assessment timelines (start of procedure, clock stop times, CHMP opinion, CHMP opinion date, total procedure time, total CHMP time), procedural information (expedited approval initiatives (AA, CMA, PRIME), OD status), and the number of SA meetings during the development of the drug. Information on company size was extracted from public websites. Companies were classified as small when they have less than 500 employees, medium-sized when they have between 500 and 5000 employees and large when a company has more than 5000 employees. In addition, for small companies we checked whether the company had the status of SME in the SME Register of the EMA. Data entry was checked by a second investigator and corrected in case of a data entry error.

## Results

In total, we identified 96 MAAs for new anticancer drugs in the EU with a CHMP opinion between January 2010 and December 2019. Table 1 summarizes some characteristics of the MAAs. Eighty-five MAAs received a positive CHMP opinion, whereas 11 MAAs received a negative CHMP opinion. Most applicants filed a MAA for an indication for the treatment of blood cancer (*n* = 31), skin cancer (*n* = 14), and lung cancer (*n* = 13). About two-third of the products were small molecules (*n* = 65), whereas one-third of the products were biotechnology-derived products (*n* = 31). Most of the applications were filed by large companies (*n* = 68), followed by medium-sized companies (*n* = 17) and small companies (*n* = 11). Six companies had the status of SME with the EMA. Seventy-eight applicants had at least one SA meeting with the EMA, whereas 18 applicants did not ask for SA. Fourteen MAAs were granted AA, 23 MAAs were granted a CMA, 40 MAAs were granted an OD, and 3 MAAs were granted eligibility to PRIME. Thirty-eight MAAs were not granted access to any of the established regulatory initiatives of the EMA, and 62 MAAs were not granted access to any expedited program (AA, CMA and PRIME).

**Table 1:** Characteristics of Marketing Authorization Applications for New Anticancer Drugs in the EU with CHMP Opinion Between January 2010 and December 2019 (*n* = 96).

Independent Variable	Number of MAAs (%)
Indication	
Blood cancer	31 (32%)
Breast cancer	9 (9%)
Colorectal cancer	4 (4%)
Gastric cancer	2 (2%)
Gastrointestinal stromal tumor	1 (1%)
Lung cancer	13 (14%)
Neuroendocrine tumors	2 (2%)
NTRK fusion-positive cancer	1 (1%)
Ovarian and peritoneal cancer	3 (3%)
Pancreatic cancer	1 (1%)
Prostate cancer	7 (7%)
Renal cancer	4 (4%)
Sarcoma	1 (1%)
Skin cancer	14 (15%)
Thyroid cancer	3 (3%)
Product type	
Biotechnology-derived	31 (32%)
Small molecule	65 (68%)
Company size	
Large	68 (71%)
Medium	17 (18%)
Small	11 (11%)
EMA SME status	6 (6%)
CHMP opinion	
Negative	11 (11%)
Positive	85 (89%)
Scientific advice requested	
No	18 (19%)
Yes	78 (81%)
CHMP opinion year	
2010	3 (3%)
2011	6 (6%)
2012	10 (10%)
2013	13 (14%)
2014	8 (8%)
2015	15 (16%)
2016	9 (9%)
2017	12 (13%)
2018	12 (13%)
2019	8 (8%)
EMA orphan designation/expedited program^a^	
Accelerated assessment (AA)	14 (15%)
Conditional marketing authorization (CMA)	23 (24%)
Orphan designation (OD)	40 (42%)
Priority medicine (PRIME)	3 (3%)
AA & CMA	3 (3%)
AA & PRIME	0 (0%)
CMA & PRIME	1 (1%)
Expedited program (AA, CMA & PRIME)	34 (35%)
No expedited program (AA, CMA & PRIME)	62 (65%)
OD/no expedited program (AA, CMA & PRIME)	27 (28%)
No OD/no expedited program (AA, CMA & PRIME)	38 (40%)

*EMA* European Medicines Agency, *NTRK* neurotrophic tyrosine receptor kinase.

^a^Numbers represent Marketing Authorization Applications where access to the regulatory initiative of the EMA was granted. Requests that were not granted or procedures that were reverted to normal were not included in these numbers. An applicant can be granted access to multiple regulatory initiatives.

### Effect of Special Regulatory Programs on Procedural Timelines

Thirty-four out of ninety-six MAAs were granted access to at least one expedited program offered by the EMA (Table 1). As shown in Table 2, access to an expedited program is not always granted. Seven applicants applied for a CMA, but their request was not granted. The EPARs of these products were evaluated for the reason a CMA was not granted. For 2 products sufficient data for a regular MA became available during the assessment. For the other 5 products the benefit-risk balance was negative. Seven applicants requested eligibility to AA, but their request was not granted. Reasons for not granting AA by the CHMP, as stated in the EPARs, are that the product is not considered of major public health interest (*n* = 5), or that the data is not sufficient to claim an unmet medical need (*n* = 2). For 12 products that were initially granted AA, the procedure was reverted to standard review timelines during the assessment. These also included the 3 products that were granted eligibility to PRIME. The reason for reverting the procedure to standard review times, as provided in the EPARs, was that there were major objections identified during the assessment (*n* = 10). For 2 products, no reason was provided in the EPAR.

**Table 2:** Number of Marketing Authorization Applications that Requested, were Granted or were not Granted Accelerated Assessment or Conditional Marketing Authorization.

	AA	CMA
Requested	33 (34%)	30 (31%)
Not granted	7 (7%)	7 (7%)
Granted	14 (15%)	23 (24%)
Reverted to standard	12 (13%)	N/A

*AA* accelerated assessment, *CMA* conditional marketing authorization.

Next, we calculated the average total procedure time of products granted a CMA or AA, and compared it with the average total procedure time of products that were not granted eligibility to any expedited program. Products that were granted both a CMA and AA were only included in the AA group. Moreover, products that were granted AA, but reverted to standard review timelines during assessment were only included in the no expedited program group. No difference was observed in total procedure time between small molecules and biotechnology-derived products for MAAs without any expedited program (supplementary Fig. 1a). As expected, total procedure time was reduced for both small molecules and biotechnology-derived products when AA was granted (from 370 days to 200 and 215 days, respectively; Supplementary Fig. 1a). For small molecules with a CMA, the total procedure time was increased with 24 days compared with small molecules without any expedited program (Supplementary Fig. 1a), whereas for biotechnology-derived products with a CMA the total procedure time was reduced with 35 days compared with biotechnology-derived products without any expedited program (Supplementary Fig. 1a).

**Fig. 1:**
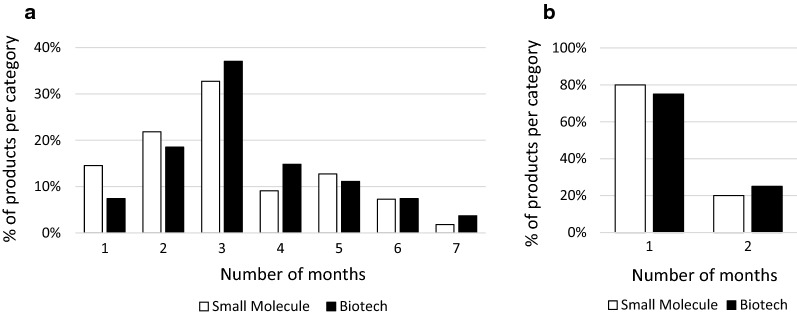
Time Required to Answer First List of Questions for Small Molecules and Biotechnology-Derived Products. **a** Number of months needed to answer the first list of questions, shown as percentage of total products per category (small molecule or biotechnology-derived product). Products that were granted accelerated assessment were excluded. **b** Number of months needed to answer the first list of questions for products granted accelerated assessment, shown as percentage of total products per category (small molecule or biotechnology-derived product).

In addition to expedited programs, anticancer drug may also benefit from other regulatory initiatives, such as OD, which offers developers protocol assistance and fee reductions. We evaluated whether the benefits offered by OD affected the total procedure time. Products that were granted access to an expedited program were excluded from the analysis. Total procedure time of small molecules with an OD was 25 days longer compared with small molecules without OD (Supplementary Fig. 1b), caused by a longer first clock stop time (data not shown). No difference in total procedure time was observed for biotechnology-derived products (Supplementary Fig. 1b).

### Time Required to Answer First List of Questions

The active assessment time of a MAA by the CHMP is interrupted by one or two clock stops, during which the applicant has to prepare answers to questions raised by the CHMP. The maximum time of the first clock stop is 3 months, but an extension of 3 months is possible if an appropriate scientific justification is given and the CHMP expects that the applicant will be able to answer all questions [13]. A clock stop longer than 6 months is normally not allowed. We showed that for small molecules 69% of the applicants and for biotechnology-derived products 63% of the applicants were able to answer the list of questions within 3 months (Fig. 1a). 29% of the applicants for small molecules and 33% of the applicants for biotechnology-derived products were able to answer the list of questions between 4 and 6 months (Fig. 1a). One applicant (2%) for small molecules and one applicant (4%) for biotechnology-derived products needed more than 6 months to answer the list of questions (Fig. 1a). For AA the maximum time of the first clock stop is 1 month [14]. 80% of the applicants for small molecules and 75% of the applicants for biotechnology-derived products were able to answer the list of questions within 1 month (Fig. 1b). Two applicants (20%) for small molecules and one applicant for biotechnology-derived products (25%) needed more than 1 month to answer the list of questions (Fig. 1b). No justification was provided in the EPAR why more time was needed or granted.

### Total Procedure Time and CHMP Opinions Over Time

Over time regulatory guidelines and procedures change to optimize procedures and provide guidance where needed to assist companies with the development of medicinal products and increase the chance of receiving a positive CHMP opinion. This may affect total procedure times and reasons for granting a negative CHMP opinion. We therefore looked at the effect of time on total procedure time and the reasons for granting a negative CHMP opinion. Products that were granted AA were not included in the analysis, as the number of products was too low to evaluate the effect of time on total procedure time. Although there was some variation in total procedure time between the years, the total procedure time did not change over the years (Supplementary Fig. 2).

Eleven medicinal products received a negative CHMP opinion between 2010 and 2019 (Table 1). The reasons for granting a negative CHMP opinion, as stated in the EPARs, were evaluated. For all products with a negative CHMP opinion there were major concerns regarding the efficacy of the medicinal product that were still unresolved at the time of the CHMP opinion, and the CHMP considered the efficacy of the medicinal products not sufficiently demonstrated (Table 3). When we looked in more detail at the major concerns regarding efficacy, we observed that between 2010 and 2015 there were mainly issues with the design of the pivotal study, which was uncontrolled (3 MAAs) or exploratory in nature (2 MAAs), whereas between 2016 and 2019 there were mainly issues with efficacy which was not considered convincing enough for applications which only contained one pivotal trial (5 MAAs). For 1 MAA, the CHMP questioned the meaningfulness of differences in the endpoints. Unresolved major concerns regarding quality and efficacy were less common but were present throughout the entire decade (Table 3).

**Table 3:** Grounds for Refusal of Marketing Authorization Applications with a Negative CHMP Opinion.

Product Name	CHMP Opinion Year	Company Size	Grounds for Refusal		
			Quality	Efficacy	Safety
Folotyn	2012	Small		x	
Istodax	2012	Large	x	x	
Masican	2013	Small	x	x	x
Masiviera	2014	Small	x	x	x
Lympreva	2015	Small	x	x	
Ninlaro	2016	Large		x	
Onzeald	2017	Medium		x	
Aplidin	2017	Medium		x	
Human IGG1 monoclonal antibody specific for human interleukin-1 alpha Xbiotech	2017	Small	x	x	x
Nerlynx	2018	Small		x	x
Vanflyta	2019	Medium		x	

*CHMP* Committee for Medicinal Products for Human Use.

### Effect of SA on Procedural Timelines

**Fig. 2:**
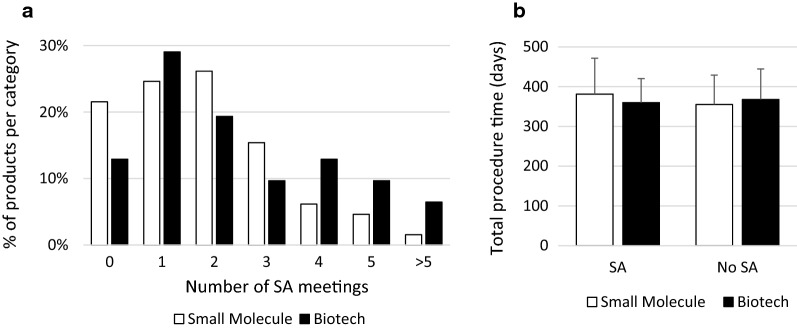
Effect of Scientific Advice on Total Procedure Time for Small Molecules and Biotechnology-Derived Products. **a** Number of scientific advice (SA) meetings companies had during the development of the medicinal product, shown as percentage of total products per category (small molecule or biotechnology-derived product). **b** Total procedure time of products for which companies had SA meetings during the development of the product compared with products for which companies did not have SA meetings. Products that were granted accelerated assessment were excluded. Values are expressed as mean ± SD.

During the development of a medicinal product, pharmaceutical companies can discuss critical issues in the drug development process with regulators in the form of SA. SA is not mandatory, but it helps pharmaceutical companies to ensure that they perform the appropriate tests and studies, so that no major objections regarding the design of the studies will be raised during the evaluation of the MAA. We found that 78 applicants had at least one SA meeting with the EMA, whereas 18 applicants did not ask for SA (Table 1). Not asking for SA was more common for developers of small molecules (*n* = 14, 22%) compared with developers of biotechnology-derived products (*n* = 4, 13%; Fig. 2a). Moreover, for biotechnology-derived products there was a trend to have multiple SA meetings (Fig. 2a). When we looked at the type of products for which no SA was asked, we observed that the majority of the products were protein kinase inhibitors. In general, most of the products for which no SA was asked were not new in their class.

Next, we evaluated the effect of having SA meetings on the total procedure time. Results for AA have not been evaluated, as there were only 3 companies which were granted AA that did not ask for SA. For products without AA, we showed that not having SA did not negatively affect the total procedure time (Fig. 2b). For biotechnology-derived products there was no difference in the total procedure time. For small molecules the total procedure time was even a little bit shorter (355 days for products without SA versus 381 days for products with SA; Fig. 2b). When we evaluated the success rate of MAAs that did not have SA, we showed that 16 out of 18 MAAs received a positive CHMP opinion. These were all from medium-sized and large companies (Table 4). Two out of eighteen MAAs received a negative CHMP opinion, these were both from small companies (Table 4).

**Table 4:** Success Rate of Marketing Authorization Applications Without Scientific Advice for Small, Medium-Sized and Large Companies.

	Total (*n*)	Medium and Large Companies (*n*)	Small Companies (*n*)
Positive CHMP opinion	16	16	0
Negative CHMP opinion	2	0	2

*CHMP* Committee for Medicinal Products for Human Use, *n* number of Marketing Authorization Applications.

### Effect of Company Size on Procedural Timelines

Small companies are an important source of innovative medicines and play a major role in the development of new medicines. Of the 96 MAAs for new anticancer drugs, 11 MAAs were for small companies (Table 1). We evaluated the effect of company size on the total procedure time. As only one small company was granted AA, for all analysis regarding company size we only focused on products that were not granted AA. We compared the total procedure time of small companies with total procedure time of medium-sized and large companies and showed that total procedure time was much longer for small companies versus medium-sized and large companies (483 days versus 356 days; supplementary Fig. 3a). This increased total procedure time is mainly caused by an increased total clock stop time and first clock stop time (supplementary Fig. 3a).

When we further compared small companies with medium-sized and large companies, we noticed that the success rate of obtaining a positive CHMP opinion was much lower for small companies. 93% of the MAAs of medium-sized and large companies received a positive CHMP opinion, whereas only 40% of the MAAs of small companies received a positive CHMP opinion (supplementary Fig. 3b). The total procedure time for small companies was not affected by the high percentage of negative opinions (supplementary Fig. 3c). Something that stood out is that for all MAAs of small companies there were major concerns regarding the clinical efficacy. When the provided evidence for clinical efficacy was considered insufficient, this resulted in a negative CHMP opinion. For some of the products with a negative CHMP opinion there were also major concerns regarding quality, pharmacodynamics, pharmacokinetics and safety.

In Table 4, we showed that two small companies that did not ask for SA received a negative CHMP opinion, while the medium-sized and large companies that did not ask for SA received a positive CHMP opinion. As small companies generally have limited knowledge of the regulation of medicines, asking for SA may be of special interest for these companies. We evaluated the effect of company size on the number of SA meetings, and showed that the small companies had maximum three SA meeting, whereas medium-sized and large companies sometimes had more SA meetings (supplementary Fig. 3d). When we compared the number of SA meetings of small companies with a positive CHMP opinion with the number of SA meetings of small companies with a negative CHMP opinion, we found that small companies with a positive CHMP opinion had more SA meetings (supplementary Fig. 3e). Moreover, when we evaluated the EPARs on compliance with SA, we showed that all small companies with a positive CHMP opinion were compliant with SA, whereas for companies with a negative CHMP opinion one company was not compliant with SA and one company was only partly compliant with SA given (Table 5).

**Table 5:** Compliance with Scientific Advice for Small Companies with a Positive or Negative CHMP Opinion.

	Compliant (*n*)	Partly compliant (*n*)	Not compliant (*n*)	No SA (*n*)
Positive CHMP opinion	4	0	0	0
Negative CHMP opinion	2	1	1	2

*CHMP* Committee for Medicinal Products for Human Use, *SA* scientific advice, *n* number of Marketing Authorization Applications.

## Discussion

In the present study, we showed that AA and company size are major determinants of total procedure time of MAAs for anticancer drugs in the EU in the period 2010 to 2019. As expected, total procedure time was reduced when AA was granted. In contrast, total procedure time was increased for small companies compared with medium-size and large companies. Total procedure time was only mildly affected by granting a CMA, an OD or having SA. In addition, we showed that about one-third of the applicants were granted access to at least one of the expedited programs of the EMA. More applicants requested access to an expedited program, but this is not always granted. Especially for AA the success rate is low, as access to AA is not always granted, and the procedure is often reverted to standard assessment timelines during the procedure. For this reason, the EMA optimized in 2016 the procedural framework of AA with the introduction of an additional list of questions and the opportunity to reach earlier opinions [6]. In addition, guidance documents were revised. The expectation of the EMA was that with the introduction of the additional list of questions the number of applications that were reverted to standard assessment times could be reduced. When we look at products that were granted access to AA and for which the procedure started after September 2016, five out of six procedures were reverted to standard assessment timelines (data not shown). This may suggest that the optimization of the procedural framework of AA did not have the expected effect. However, three years may be too short to already see the expected effect. Moreover, anticancer drugs are not the only category of drugs for which AA can be granted, and other categories of drugs should be evaluated as well to say something about the success of the optimization of the procedural framework of AA.

Three of the applications for which AA was reverted to standard assessment timelines were granted eligibility to PRIME. PRIME was launched in March 2016 by the EMA to enhance support for the development of medicines that target an unmet medical need [9]. It is based on increased interaction and early dialogue between developers and EMA to optimize development plans and speed up evaluation so that these medicines can reach patients earlier. To be accepted, a medicine has to show its potential to benefit patients with unmet medical needs based on early clinical data. Medicines that benefitted from PRIME can be expected to be eligible for AA. In total three anticancer drugs that received a CHMP opinion between 2010 and 2019 were granted eligibility to PRIME. All three products were granted eligibility to AA, but the procedure was reverted to standard timelines for all three products during the assessment. PRIME is still new, and only the first products granted eligibility could be analyzed in our study. More time is needed to evaluate the effectiveness of PRIME.

In our study, we showed that granting a CMA affected the total procedure time only mildly. Total procedure time was longer for small molecules, but shorter for biotechnology-derived products. A previous study showed that total procedure time of anticancer drugs which were granted a CMA in the period 2006–2013 was longer compared with anticancer drugs which were granted a full marketing authorization [15]. A limitation of our study is that we only assessed the total procedure time. It is expected that a CMA mainly affects the total development time, as granting of a marketing authorization is based on ‘less than comprehensive clinical data’. The study of Hoekman et al., however, did not show a reduced development time when products were granted a CMA, as companies did not use a CMA as a prospectively planned pathway to obtain early access, but rather as a rescue option when submitted data were not strong enough to justify a full marketing authorization.

An interesting observation of our study is that the majority of the anticancer drugs (two-third) did not use or qualify for any of the early access tools offered by the EMA. There may be an expectation in the industry that anticancer drugs would always reach the market as soon as possible and would easily qualify for regulatory initiatives of the EMA to expedite the development and authorization of the drugs. Our study highlights that this is not the case, and highlights that the EMA does not see all anticancer drugs fulfilling an unmet medical need.

Regulatory authorities like the EMA continuously evaluate regulatory initiatives for their effectiveness, and if needed optimize procedures and guidelines to support companies in the development of medicinal products and increase the chance of receiving a positive CHMP opinion. Despite potential optimizations during the years, average total procedure time and the number of negative CHMP opinions were not changed in the past decade. With regards to total procedure time, the review by EMA is taking most of the total procedure time and the response time by applicant had only a limited contribution to the total procedure time. It must be stressed that the EMA follows the legal review times and therefore only with special initiatives (AA) it would be possible to reduce the total review time significantly. Major concerns regarding clinical efficacy were present for all MAAs with a negative CHMP opinion over the past decade, but shifted from issues with the design of the pivotal study, which were uncontrolled or exploratory in nature, to issues with efficacy which was not considered convincing enough for applications which only contained one pivotal trial. This trend should, however, be confirmed in a larger study, as the number of negative CHMP opinions in this study is low. Major concerns may potentially be addressed during the procedure through clarifications and additional analysis. However, when the appropriate control groups are missing in the pivotal trial or convincing data cannot be provided for the pivotal trial, it will be very difficult to address the major concerns during the assessment. Issues could, however, be prevented by having SA prior to the application, where the acceptability of the design of the pivotal trial or data of the pivotal study could have been discussed.

Next to AA, the total procedure time of MAAs for anticancer drugs was mainly affected by company size. Our study showed that total procedure time of small companies is much longer compared with medium-sized and large companies, which was mainly caused by an increased clock stop time. In addition, the success of small companies in obtaining a positive CHMP opinion is much lower compared with medium-sized and large companies. From EPARs it is not clear why small companies need more time to answer questions compared with medium-sized and large companies. It is, however, known that companies resolve major concerns through clarifications, additional analysis and providing supplementary data that become available during the procedure [16]. In addition, medium-sized and large companies often have agency response teams which identify potential weaknesses of the registration file and initiate additional studies. Providing the information requested by the CHMP in a timely manner is particularly challenging for small companies, as they often have insufficient human resources and therefore may need more time to answer questions. Small companies also often have limited financial resources, which may hamper drug development and regulatory compliance. In addition, small companies often need to source out several activities to contract manufacturing organizations or contract research organizations and these companies also need to be aligned in the agency response process.

A recent study analyzed major concerns of MAAs of SMEs and their impact on outcomes between 2011 and 2015 [16]. They showed that 66% of the total 64 applications had a positive CHMP opinion. Major objections were mainly observed in quality (73%), clinical efficacy (80%) and clinical safety (48%). Major objections associated with a negative CHMP opinion were mainly related to the choice of endpoints, clinical safety concerns and pharmacodynamics and pharmacokinetics. In our study major concerns regarding clinical efficacy were present for all MAAs with a negative CHMP opinion, but for small companies this was often accompanied with concerns regarding quality, pharmacodynamics, pharmacokinetics and safety. Based on the available data and the low number of small companies in our study we cannot specify which factor influenced the CHMP outcome of small companies most in our study.

Major objections during the assessment of the MAA could have been prevented by having SA during the development of the drug. We showed that a comparable percentage of small companies had no SA meetings, one SA meeting or two SA meetings compared with medium-sized and large companies. However, where small companies had maximum three SA meetings, medium-sized and large companies sometimes had more SA meetings. Small companies with a positive CHMP opinion had more SA meetings and were more compliant with SA given than small companies with a negative CHMP opinion. This is in line with a previous study that showed that obtaining and especially adhering to SA is important for companies in obtaining a positive CHMP opinion [17].

There were some limitations to this study. First, our research was based on the information that is made publicly available by the EMA in the form of EPARs. EPARs only contain a summary of the full dossier and our data are therefore dependent on the information summarized and published by the EMA. A second limitation of the study is that we did not include MAAs that were withdrawn by the applicant. Especially medium-sized and large companies may decide to withdraw their application when they receive major objections. Based on their experience they may know better when they will not be able to resolve major objections in the timeframe given. This may have biased the success rate of medium-sized and large companies. A final limitation of our study is that the number of cases is relatively low. Especially the number of MAAs of small companies and the number of MAAs with a negative CHMP opinion were low. However, it must be recognized that negative opinions are not seen frequently and there are few small companies which succeed in bringing a new product to the market. Therefore, the low numbers probably represent the best available dataset.

Our study provides an early and limited analysis of the success of the recent optimization of the procedural framework of AA and the introduction of PRIME by the EMA. Our study highlights that in the first years after the procedural optimization of AA the intended goal of the EMA, i.e., reducing the number of applications that were reverted to standard assessment time, was not met for anticancer drugs. Moreover, although the three products that were granted PRIME qualified for AA, the procedure was reverted to standard assessment time for all three products. It may take some time to see the intended effects, but the EMA is advised to continue to monitor the success of early access tools and further optimize when needed. Developers of anticancer drugs should keep in mind that developing anticancer drugs does not necessarily mean that a product qualifies for an early access tool offered by the EMA. Early access tools are only for products that target an unmet medical need, which means a condition for which there exists no satisfactory treatment, or where the medicinal product will be of major therapeutic advantage. To increase the chance of success, developers are highly advised to comply with regulatory guidelines and seek SA if there are no specific guidelines or if they want to deviate from the regulatory guidelines.

## Conclusion

Taken together, our study shows that the total procedure time of MAAs for anticancer drugs in the EU in the period 2010 to 2019 is mainly affected by the granting of AA, which reduced the total procedure time, and company size, where total procedure time is much longer for small companies. Moreover, our study showed that, while for medium-sized and large companies, it is not always needed to have SA, for small companies it is highly advised to have SA, and especially to adhere to the advice given in the SA meeting.

## Supplementary Information

Below is the link to the electronic supplementary material.

Electronic supplementary material 1 (PDF 103 kb)

Electronic supplementary material 2 (PDF 106 kb)

Electronic supplementary material 3 (PDF 214 kb)
