# Supplementary material for: A Decade of Marketing Authorization Applications of Anticancer Drugs in the European Union: An Analysis of Procedural Timelines
Source: Ther Innov Regul Sci. 2021 Feb 4;55(4):633–42. doi: 10.1007/s43441-021-00260-5 (PMC8238922; doi:10.1007/s43441-021-00260-5)
Supplement: Supplementary file 1 — Electronic supplementary material 1 (PDF 103 kb) [file 43441_2021_260_MOESM1_ESM.pdf]

Supplementary figure 1

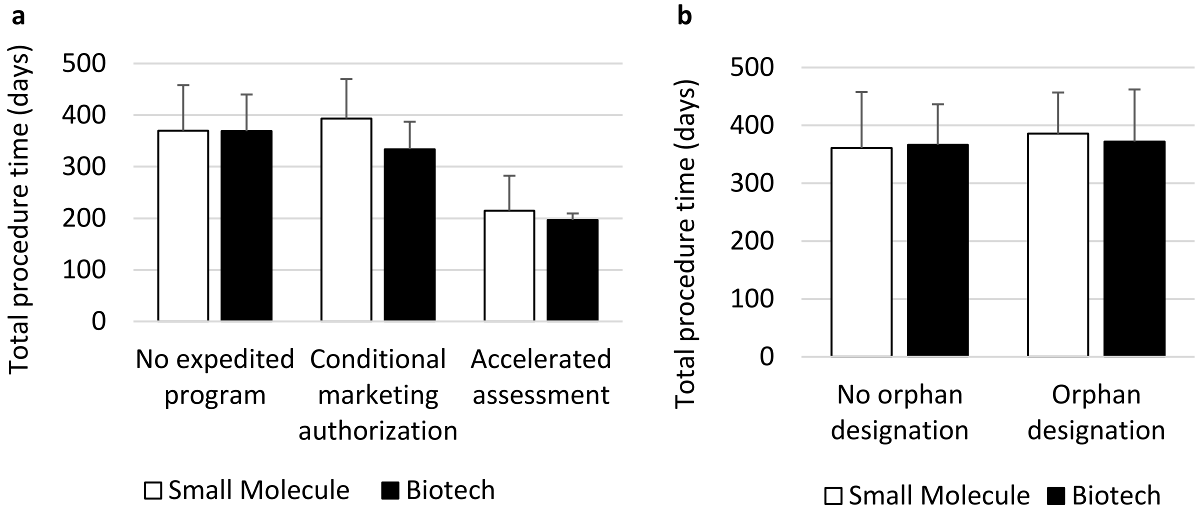

Supplementary figure 1: Effect of special regulatory programs on total procedure time for small molecules and biotechnology-derived products. a) Total procedure time of products granted with a conditional marketing authorization (CMA) or accelerated assessment (AA) compared with total procedure time of products without any expedited program. **b)** Total procedure time of products granted with an orphan designation compared with products without an orphan designation. Products that were granted a CMA or AA were excluded. **a-b)** Values are expressed as mean ± SD.

Article title: A decade of marketing authorization applications of anticancer drugs in the European Union: an analysis of procedural timelines
Journal name: Therapeutic Innovation & Regulatory Science
Author names: Marjolein Garsen[1], Maaike Steenhof[1], Alex Zwiers[1]
Affiliation: [1]Zwiers Regulatory Consultancy, Oss, the Netherlands
Email address of the corresponding author: Marjolein.Garsen@az-regulatory.com
