# Supplementary material for: A Decade of Marketing Authorization Applications of Anticancer Drugs in the European Union: An Analysis of Procedural Timelines
Source: Ther Innov Regul Sci. 2021 Feb 4;55(4):633–42. doi: 10.1007/s43441-021-00260-5 (PMC8238922; doi:10.1007/s43441-021-00260-5)
Supplement: Supplementary file 2 — Electronic supplementary material 2 (PDF 106 kb) [file 43441_2021_260_MOESM2_ESM.pdf]

Supplementary figure 2

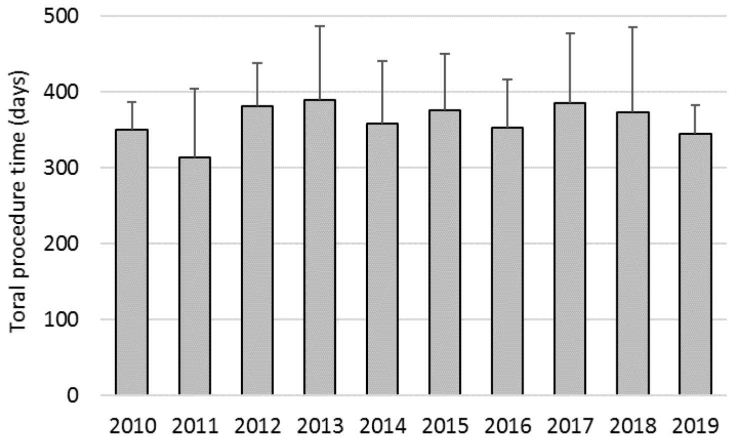

Supplementary figure 2: Total procedure time per year from 2010 to 2019. Values are expressed as mean ± SD.

Article title: A decade of marketing authorization applications of anticancer drugs in the European Union: an analysis of procedural timelines

Journal name: Therapeutic Innovation & Regulatory Science

Author names: Marjolein Garsen[1], Maaike Steenhof[1], Alex Zwiers[1]

Affiliation: [1]Zwiers Regulatory Consultancy, Oss, the Netherlands

Email address of the corresponding author: Marjolein.Garsen@az-regulatory.com
