# Supplementary material for: A Decade of Marketing Authorization Applications of Anticancer Drugs in the European Union: An Analysis of Procedural Timelines
Source: Ther Innov Regul Sci. 2021 Feb 4;55(4):633–42. doi: 10.1007/s43441-021-00260-5 (PMC8238922; doi:10.1007/s43441-021-00260-5)
Supplement: Supplementary file 3 — Electronic supplementary material 3 (PDF 214 kb) [file 43441_2021_260_MOESM3_ESM.pdf]

Supplementary figure 3

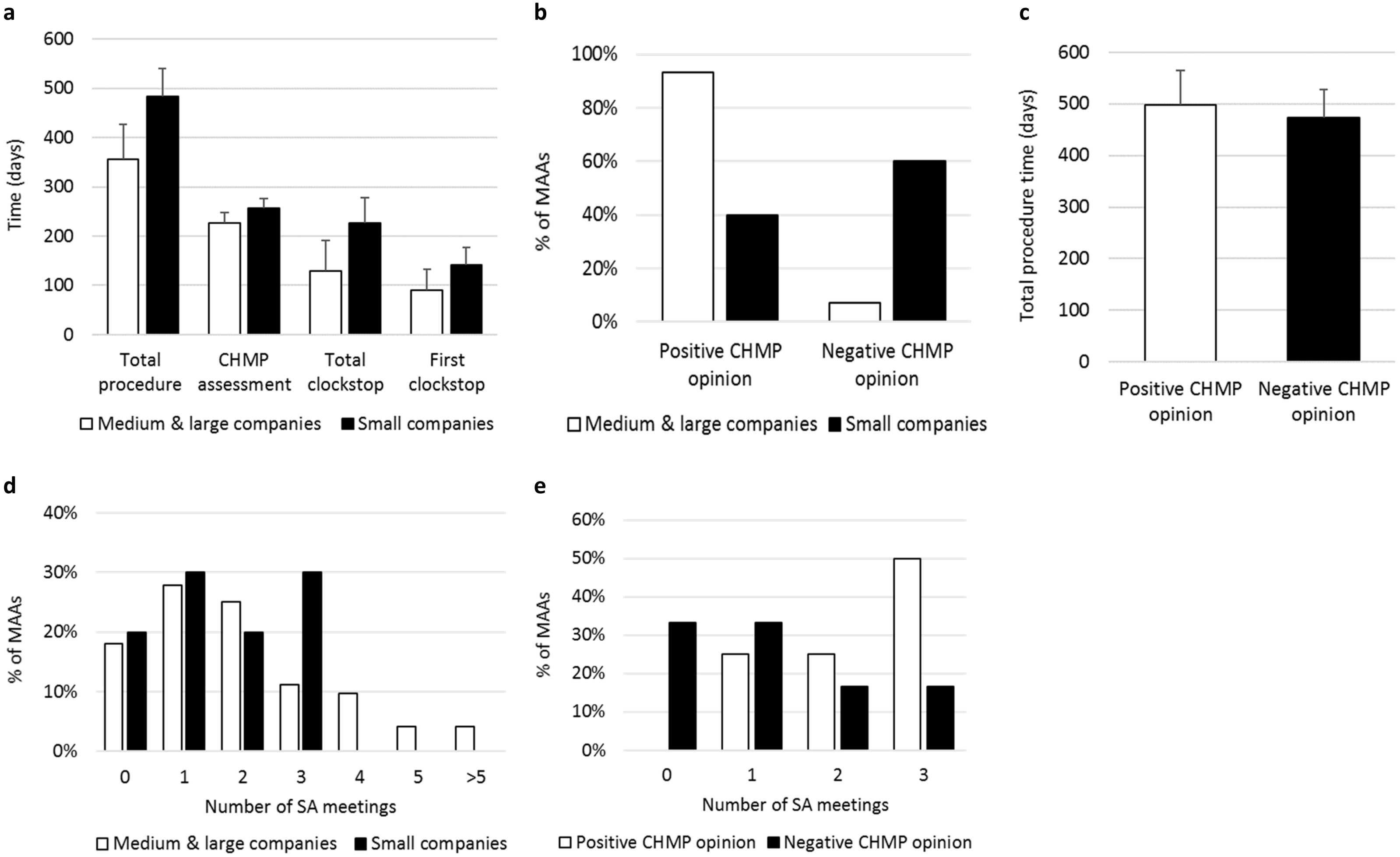

Supplementary figure 3: Effect of company size on total procedure time. a) Total procedure time, CHMP assessment time, total clock stop time and first clock stop time of medium-sized and large companies compared with small companies. **b)** Percentage of marketing authorization applications (MAAs) that were granted a positive CHMP opinion or a negative CHMP opinion for medium-sized and large companies compared with small companies. **c)** Total procedure time of MAAs of small companies with a positive CHMP opinion compared with MAAs of small

companies with a negative CHMP opinion. **d)** Number of scientific advice (SA) meetings companies had during the development of the medicinal product, shown as percentage of total MAAs of medium-sized and large companies or small companies. **e)** Number of scientific advice (SA) meetings small companies had during the development of the medicinal product, shown as percentage of total MAAs of small companies with a positive CHMP opinion or negative CHMP opinion. **a-e)** Products that were granted accelerated assessment were excluded. **a,c)** Values are expressed as mean ± SD. CHMP: Committee for Medicinal Products for Human Use.

Article title: A decade of marketing authorization applications of anticancer drugs in the European Union: an analysis of procedural timelines
Journal name: Therapeutic Innovation & Regulatory Science
Author names: Marjolein Garsen[1], Maaike Steenhof[1], Alex Zwiers[1]
Affiliation: [1]Zwiers Regulatory Consultancy, Oss, the Netherlands
Email address of the corresponding author: Marjolein.Garsen@az-regulatory.com
